# 

**Published:** 1881-01

**Authors:** 


					﻿WholH Number,
CCCCXXXIII.
January, 1881.
Number t,
Vol. XLII.
THE
CHICAGO
MEDIC AL
T ournal and Examiner
EDITORS:
N. S. DAVIS. M.D., LL.D. JAS. NEVINS HYDE, A.M., M.D.
DANIEL R. BROWER, M.D.
CHICAGO:
PUBLISHED BY THE CHICAGO MEDICAL PRESS ASSOCIATION,
188 Clark Street.
£g“Read the Notice of this Journal among Advertisements.
				

